# Should we all just take 10? A meta-analysis of wakeful rest

**DOI:** 10.3758/s13423-025-02778-3

**Published:** 2026-01-15

**Authors:** Daniela Parra, Zhiyong Zhang, Gabriel Radvansky

**Affiliations:** Department of Psychology, University of Notre Dame, Notre Dame, IN USA

**Keywords:** Wakeful rest and memory, Post-encoding rest, Minimal-interference and memory

## Abstract

According to previous research, if people rest quietly for a brief period of time after learning, they have better memory (i.e., reduced forgetting) after a delay compared to when they engage in a cognitively demanding task. We call this the *wakeful rest effect*. It has been observed with different kinds of study items, interference tasks, and delay intervals involving younger adults, older adults, and patients with amnesia. Despite the sometimes-presumed robustness of the effect, many studies have failed to observe significant results, particularly in healthy young adult populations. This random-effects meta-analysis combined 142 effect sizes from 51 studies to evaluate the evidence for the wakeful rest effect and to identify the sources of variation. Meta-regression was also done. As expected, there were larger effects for patient populations than for healthy populations, as well as weaker effects for younger than older adults. The results of this meta-analysis can inform further research on the potential benefits of wakeful rest.

## Introduction

**Fig. 1:**
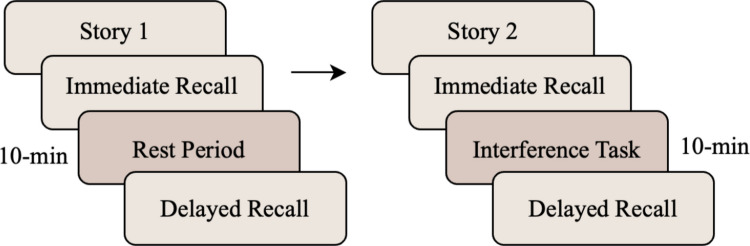
Example procedure for testing the wakeful rest effect

Emerging behavioral studies suggest that some memory consolidation processes that are active during sleep may also be active during wakeful rest. The *wakeful rest effect* refers to better memory performance (i.e., reduced forgetting) when people are given a period of wakeful rest after learning (see, e.g., Cowan et al., 2004; Dewar et al., 2009). The paradigm usually involves presenting people with learning material, such as lists of nouns, and then immediately testing their memory for it (Fig. 1). This immediate test is intended to discourage further rehearsal of the information because people may think that the need to remember the information has now passed. In addition, the immediate test provides a baseline of post-encoding retention. Following the immediate test, people either rest quietly or complete an interference task for a brief period of time. While not all studies require people to close their eyes during the rest period (e.g., Craig et al., 2014), some studies involve instructions to do so (e.g., Martini et al., 2020a). People are then given a second memory test. Typically, these studies use a repeated-measures design, so that the same people undergo both interference and wakeful rest conditions.

Here, we first introduce the consolidation theory for the wakeful rest effect and review existing studies across patient populations, age groups, and a range of study characteristics. Next, we detail the procedure for the literature search, data extraction, and analysis for a random-effects meta-analysis. After that we report the results of the analysis and conclude with a discussion of the results, limitations of the current procedure, and potential future steps.

## Wakeful rest and consolidation

Consolidation is the process by which memory traces are stabilized over time (Dudai, 2004). It is often suggested that a primary mechanism underlying the two major types of consolidation (synaptic and systems) is repeated neural reactivation. During synaptic consolidation, the pattern of neurons that was activated at encoding is reactivated within the hippocampus (McNaughton & Morris, 1987; Mednick et al., 2011). This reactivation results in neural plasticity that solidifies synaptic connections between neurons that make up a memory trace. Alternatively, systems consolidation is the gradual reorganization of a memory trace, whereby a memory trace is integrated in distributed cortical regions.

As summarized by Wamsley (2022), numerous mechanisms that are associated with neural reactivation during sleep are thought to support consolidation. Sharp-wave ripples (SWRs), for instance, co-occur with hippocampal reactivation and seem to be related to memory performance (Kudrimoti et al., 1999). In rodents, blocking SWRs hinders memory (Ego-Stengel & Wilson, 2010); in humans, ripples predict later memory (Axmacher et al., 2008). While traditionally linked to sleep, a growing body of research suggests that some of these mechanisms are also operating during wakeful rest. For instance, reduced levels of acetylcholine were present during wakeful rest in cats, in contrast to active wake conditions (Marrosu et al., 1995). Moreover, neural reactivation and SWRs have been observed in humans during both sleep and wakeful rest (Chen et al., 2021; Hermans et al., 2017; Schreiner et al., 2024). Importantly, other mechanisms thought to support consolidation, such as sleep spindles, are unique to sleep (Mednick et al., 2013). To date, no causal link has been established between sleep spindles and memory, but it may be that sleep spindles target an aspect of consolidation that is not available during wakeful rest. Nonetheless, behavioral studies suggest that there are comparable memory benefits after post-encoding sleep and wakeful rest (e.g., Wang et al., 2021).

A memory benefit from post-encoding sleep is well established (Stickgold, 2005), but the extent to which wakeful rest also enhances memory remains unclear. Therefore, the following review aims to specifically focus on the memory consequences of wakeful rest.

### Study populations

It is well known in the literature that there are large quantitative and qualitative differences in memory across patients with amnesia, healthy older adults, and healthy younger adults. For example, people with anterograde amnesia show much faster forgetting than older adults (Ally et al., 2013; Sadek et al., 2004), and older adults show much faster forgetting than younger adults (Fraundorf et al., 2019). As such, we decided to treat these groups separately.

#### Patients with amnesia

Unlike many lines of study on memory that began with normal populations, contemporary work on wakeful rest began with patients with anterograde amnesia. Cowan et al. (2004) hypothesized that reductions in retroactive interference could yield a benefit in memory for people with weakened or damaged abilities. They compared the recall performance of six patients with anterograde amnesia (of various etiologies) and six age-matched controls. People were auditorily presented with lists of words and then either completed psychometric tasks (interference) or rested quietly in a dark room (wakeful rest). Both patients and controls had better performance when they rested quietly, regardless of whether the researchers used a 10-min or 1-h rest period, or whether they used lists of words or stories. This highlighted the generality and strength of their results. While it is believed that many patients with severe anterograde amnesia forget events within a minute, Cowan et al.’s results support the idea that this forgetting is not due to the passage of time, per se, but to the disruption of memory consolidation processes by interfering stimuli.

Cowan et al. (2005) did similar studies with patients with mild cognitive impairment (MCI), in which people were verbally presented with four stories, two of which were followed by a wakeful rest period and the other two by a period of psychometric tasks. They found that both controls and patients displayed better memory under minimal interference compared to psychometric tasks. Another study with patients with amnestic MCI by Dewar et al. (2009) assessed whether delaying interference, not just the absence of it, still yielded a benefit. All participants were given an interference-producing task (picture-naming) at varying onset times: immediately, 3, or 6 min into a 9-min delay interval, in addition to an unfilled retention interval. Patients, but not healthy controls, retained more information when the task occurred 6 min into the delay than when it occurred immediately or 3 min into the interval.

Across studies, patients typically had a variety of causes for their anterograde amnesia, including, but not limited to, embolic strokes, closed-head injuries, anoxia, and Korsakoff syndrome. These results have been replicated multiple times in patients with other types of amnesia as well as different kinds of memory loss (Alber et al., 2014; Dewar et al., 2010, 2012b). For instance, Dewar et al. (2012b) showed that both patients with amnestic MCI and those with mild to moderate Alzheimer’s disease benefitted from a period of wakeful rest. Alber et al. (2014) showed that this benefit can last for at least a week after learning, which is remarkable given that anterograde amnesia is characterized by an inability to retain new information over extended periods of time. Importantly, this week-long benefit may last for some kinds of memory deficits, but not for others. Evans et al. (2021) found that while this effect was observed in stroke survivors when they were tested 15–30 min after the presentation of the materials, no benefit was seen after a week, although a benefit was observed for healthy older adults. McGhee et al. (2020) also reported confusing results, because a rest benefit in patients occurred only when encoding was preceded by a spot-the-difference task (as opposed to an unfilled pre-encoding task).

Although the study of wakeful rest and memory began with the study of patient populations, it has subsequently also been used to study groups of healthy people. We consider this in the next sections.

#### Older adults

The wakeful rest effect has also been observed with normally aging adults (Dewar et al., 2012a, 2014). Dewar et al. (2012a) tested older adults who scored normally on an extensive neuropsychological battery, which assesses five cognitive domains, including memory. People underwent two counterbalanced trials, one in which they rested quietly for 10 min after the presentation of a story, and another in which they completed a spot-the-difference task for the same amount of time. They were tested immediately, 15–30 min, and a week after the story presentation. People did better when they rested quietly with the benefit lasting a week later. These results could not solely be attributed to test effects from the first delayed test because researchers did a second experiment without it and observed similar outcomes. These results, along with the Alber et al. (2014) study involving people with memory deficits, showed support for the long-term benefit of a period of quiet rest. While Alber et al. observed long-term results in both patients and older adults, benefits were only present for older adults in Evans et al. (2021).

The wakeful rest effect in older adults has been replicated using between-subjects designs and with recognition tests (Dewar et al., 2014; Martini et al., 2019b), suggesting that impaired memory is not a requisite for a rest-related benefit in memory for older adults. Despite this, Dewar et al. (2009) previously mentioned findings suggested that a 6-min rest only improves memory for patients and not older adults, which may indicate that while 6 min may be sufficient to aid memory for those with anterograde amnesia, delaying interference may not yield a benefit in people with normal memory. Nonetheless, forgetting was reduced for both patients and controls in the completely unfilled condition, with patients displaying a greater benefit from minimized interference.

#### Young adults

Researchers have also studied the wakeful rest effect on young adults and children. However, the results are inconsistent (Craig et al., 2014, 2015, 2016a; Martini et al., 2019a). In a study comparing healthy younger and older adults, Sacripante et al. (2019) found that the groups did not differ in the overall benefit. In another study investigating spatial memory, people either rested or underwent an unrelated spot-the-difference task after learning a long route in a virtual reality environment. Craig et al. (2016b) found that both age groups had a comparable decrease in angle of absolute pointing error in a cognitive map test. This was replicated by Craig et al. (2016a) for young adults. However, when the cognitive map test was scored based on direction judgments, as in the Craig et al. (2015) study, young adults performed at ceiling after the retention delay. They also performed at ceiling when freely recalling landmarks, and so could not show the rest benefit that older adults did (Craig et al., 2016b).

Despite these mostly positive findings, Dewar et al. (2010) failed to observe a wakeful rest effect in healthy controls whose ages ranged from 21 to 74 years,^1^ even though it was observed in ten patients with a similar age range. This could have been a result of near-ceiling performance in controls, lack of power, or the lack of control for age, although Varma et al. (2017) were also unable to find a significant effect of rest in young adults across six different experiments. An unpublished study by Carlson (2018) showed no difference in the wakeful rest condition versus the commonly used spot-the-difference task. Note that people were asked to do a breathing task using a phone app during the rest condition, which may have acted as a source of interference and prevented consolidation.

Interestingly, Martini et al. (2020b) found null effects in young adults when comparing performance after a rest period with that after an attention and concentration task (d2), unless working memory span scores were included as a covariate. A brief period of rest improved memory to a greater extent for people with high working memory span scores. The distractor task disrupted memory more for these people than those with lower working memory span scores. The authors suggested that people with higher working memory span scores created stronger associations at encoding, which led to more efficient consolidation at rest. They might have also been more engaged in the d2 task than those with lower scores. The null effect found by some studies could be a result of a lack of power, measurement error, or heterogeneity in participant age or testing procedures (Humiston et al., 2019).

#### Children

Brief periods of rest, compared to watching short, animated films, boosted memory for children between the ages of 10 and 13 years (Martini et al., 2021). This was also observed with 13- to 14-year-old children when compared to a problem-solving task, although the effect was qualified by retention in the immediate recall test (Martini et al., 2019a). In other words, the effect was only beneficial for children who recalled less than 43% of the words immediately after encoding. Another study showed that while children benefited from wakeful rest, adults with an age range of 18–61 years did not (Fatania & Mercer, 2017). Thus, wakeful rest may aid those with weaker memory processes, including children, older adults, and memory disordered people.

### Study characteristics

In addition to observing the wakeful rest effect across age groups with and without memory deficits, researchers have found an effect of wakeful rest on memory using different methods, including different study items, interference tasks, delay intervals, and memory tests.

#### Learning and testing

While the wakeful rest effect was first observed with study items such as words and stories, it has also been observed using photographs of everyday items, navigation routes, face/name pairs, and more (Craig & Dewar, 2018; Craig et al., 2015; Dewar et al., 2014). It is typically studied using verbal recall memory tests, but Dewar et al. (2014) showed that the effect can also be observed using recognition. Similarly, Mercer (2015) replicated this effect using cued recall of English translations of Icelandic words.

The wakeful rest effect is not specific to verbal material and may contribute to improvements in different types of declarative memory (Dewar et al., 2014; Mercer, 2015). Further support for this idea is revealed in a study by Craig et al. (2015) that examined memory for navigation routes, in which an improvement in spatial associative memory was seen, in addition to better performance for temporal-order information. Moreover, Craig and Dewar (2018) found a rest-related memory improvement using picture recognition, which targeted memory for fine details of the images. Memory for detail was also tested by Sacripante et al. (2019), who found that wakeful rest benefited older and younger adults for both gist and detail memory. Wakeful rest effects have also been observed for procedural memory tasks (Wang et al., 2021).

Resting has even impacted directed forgetting (Schlichting & Bäuml, 2017). For list-based directed forgetting, people were told to forget certain word lists and remember others, and then asked to listen to music, look at pictures, complete a counting task, or complete a calculation task. Although people successfully forgot words in the to-be-forgotten group of the distractor conditions (counting and calculations), they remembered both groups of words at the same rate in passive rest conditions (music and pictures). When asked to remember study items, wakeful rest did not aid retention. It is currently unknown how passive listening of neutral music or looking at pictures compares to sitting quietly, or whether these two activities constitute wakeful rest.

#### Interference tasks

A variety of interference tasks have also been used as a contrast to wakeful rest. Wakeful rest resulted in less forgetting compared to picture-search and cued autobiographical thinking tasks, but not compared to listening to meaningless sounds (Craig et al., 2014). In this case, meaningless sounds might not be engaging attention as the other tasks would, thereby enabling consolidation. Completing a visuospatial problem-solving task resulted in worse memory than resting (Martini et al., 2019a). Even using social media brought about poorer memory in comparison to passive rest (Martini et al., 2020a). These results are notable because they point to the ecological validity of the effect. Wakeful rest has preserved memory compared to interference tasks that involve watching movies, spot-the-difference tasks, psychometric tests, and more in verbal and spatial modalities (Cowan et al., 2004; Martini et al., 2021; Sacripante et al., 2019). Nevertheless, Varma et al. (2018) and Martini et al. (2020b) could not find the effect using an n-back and d2 task, respectively, which could indicate that only hippocampal-dependent tasks cause interference to declarative memory.

#### Delay interval

The wakeful rest effect has been observed with rest periods as short as a few minutes to as long as an hour (Cowan et al., 2004; Dewar et al., 2009), although a common rest period is about 8–12 min long. In one study, results were not present immediately after the rest versus interference period, but instead emerged 7 days after encoding (Martini et al., 2018), suggesting consolidation processes at work. While this effect has repeatedly been shown to last up to 7 days (Alber et al., 2014; Dewar et al., 2012a, 2014; Martini et al., 2019a), other studies fail to observe a 7-day benefit (Craig et al., 2015; Martini et al., 2018, 2020b).

In sum, the wakeful rest effect has repeatedly been observed with a variety of study characteristics in younger adults, older adults, and patients with amnesia. The effect has been shown to target different aspects of memory, including verbal, spatial, detail, and gist memory. As has been discussed, however, many contradictory or null results have also been observed.

### Alternative explanations

The primary theoretical account of the wakeful rest effect has been consolidation theory. Again, the idea is that periods of wakeful rest allow for memory consolidation processes to operate on newly learned information, thereby resulting in better memory following wakeful rest than a cognitively engaging activity. Despite this, there have been other explanations. The rehearsal and temporal distinctiveness accounts are considered here.

#### Rehearsal

Rehearsing material may explain the increase in memory. It may be that people remember more because they are actively rehearsing the information during the wakeful rest period. However, a large body of evidence suggests that this explanation is insufficient. First, retention intervals can be up to an hour long. Thus, continually rehearsing verbatim would be difficult, particularly in experiments using sentences or stories as memoranda, and especially for patient populations (Cowan et al., 2004, 2005). Second, the wakeful rest effect has been observed in many studies using materials that are near-impossible to rehearse, including scrambled words and routes (Craig et al., 2016a, 2016b; Dewar et al., 2014). This suggests that even if some people actively rehearse during the rest period, it is not the major player here.

#### Temporal distinctiveness

Consolidation theory states that the memory benefit is due to active consolidation processes during rest. However, another possibility is that it is due to interfering memory traces resulting from non-rest conditions. If so, it would not be the rest that aids memory, but the interference tasks that worsen it via proactive and retroactive interference processes (Underwood, 1957).

While reducing retrieval interference likely plays a role, it is unlikely that this is the main driver of the effect. In one study, pre- and post-encoding intervals were either both unfilled, both filled, or one was filled while the other was not (McGhee et al., 2020). For patients, at least one unfilled interval sufficiently blocked interference, even when the unfilled interval occurred before encoding. This suggests that reducing retrieval interference, as opposed to allowing post-encoding consolidation to occur, enhances memory. However, there were no differences for healthy participants across conditions. Thus, these results could reflect measurement error. Alternatively, the fact that a benefit was observed when the rest preceded learning is consistent with a role for some form of temporal distinctiveness. According to the temporal distinctiveness theory, resting improves memory by making the memory trace more distinct, thereby increasing the likelihood it will be retrieved. McGhee’s results suggest that this may be more relevant with patients with memory deficits, especially since healthy adults did not benefit from pre-encoding rest. Despite this, pre-encoding rest did not improve memory for patients or healthy adults across an unpublished set of experiments by McGhee (2020). Moreover, active neural processes, as previously mentioned, have been observed during wakeful rest, suggesting consolidation at work (Wamsley, 2022).

### Present study

Given the lack of consistency across studies, this meta-analysis combined effect sizes from many experiments to examine the effect of wakeful rest on memory retention. This has been done before (Humiston et al., 2019). This prior analysis found a small but significant effect of wakeful rest. However, only 12 studies were used and they were limited to healthy populations. More importantly, the analysis did not distinguish between age groups. The potential influence of age was not accounted for, leading to unexplained variance.

The present analysis aimed to expand on the previous work by improving the precision of the effect size. First, this was done by including many more studies. Additionally, the current analysis sought to determine if the relationship between rest and memory was moderated by factors such as group differences (i.e., age and amnesia). Based on the limited number of studies observing a rest benefit for young adults, we expected to find stronger effects for patients with amnesia and older adults. In addition, meta-regression was carried out as an exploratory analysis to investigate moderation effects of other study characteristics, which may explain variation across studies.

## Method

All analyses were done in R version 4.3.3 (R Core Team, 2024), primarily with the packages *meta* (v7.0–0; Balduzzi et al., 2019), *metafor* (v4.6.0; Viechtbauer, 2010), and *dmetar* (v0.1.0; Harrer et al., 2019). This study was performed using Harrer et al. (2021) as a guide.

### Eligibility criteria

The following criteria were used for a study to be considered for inclusion:

1. The study was published in or translated to English.
2. The study included a behavioral component and investigated declarative memory.
3. The study included human participants, as opposed to animal studies.
4. The study included a period of minimal-interference directly following learning or immediate testing and was compared to a similar condition with interference instead of rest.

Studies were excluded if:

1. Participants were instructed to attend to any form of stimuli during the rest period.
2. Participants were instructed to sleep during the rest period.
3. The study did not compare the rest period with an interference task.

### Literature search

**Fig. 2:**
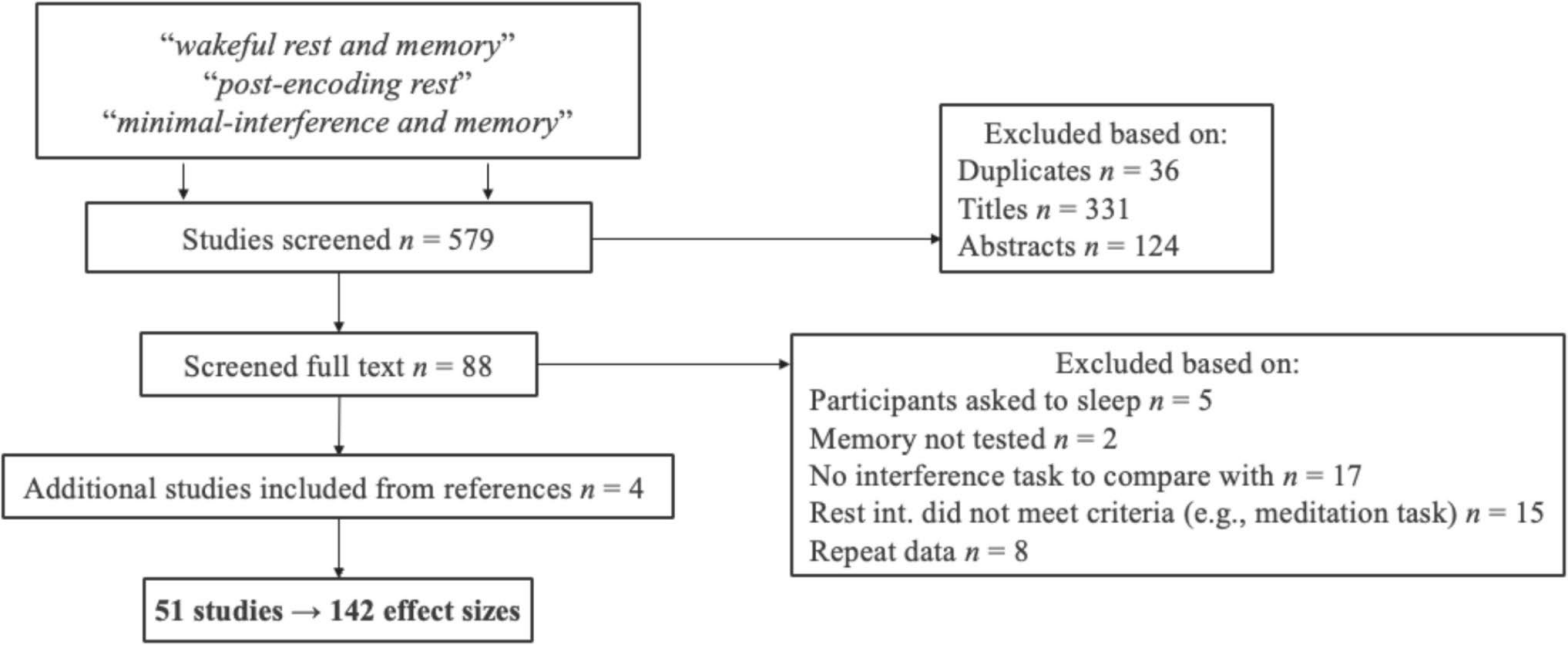
Literature search process

The literature search for studies was done using Google Scholar with keywords such as wakeful rest and memory, post-encoding rest, and minimal-interference and memory. We selected these terms because they seem to appear most often across publications. We searched the first 200 citations that appeared for each key term (spot-checking papers after this; we did not find any relevant studies past this). After screening titles, abstracts, and, subsequently, the full text (Fig. 2),^2^ the references of the acquired studies were searched and any study that met the criteria was included. Any relevant study that was encountered incidentally was also included. This resulted in a total of 142 effect sizes from 51 studies.

### Data extraction

The effect size of interest was the standardized mean difference in memory between post-rest and post-interference experimental conditions. Specifically, to calculate Cohen’s *d*, we used ${d}_{w}= \frac{{\mathcal{X}}_{rest}-{\mathcal{X}}_{int}}{\sqrt{\frac{{ s}_{rest}^{2}+{ s}_{int}^{2}}{2}}}$ for within-subject and ${d}_{b}= \frac{{\mathcal{X}}_{rest}-{\mathcal{X}}_{int}}{\sqrt{\frac{\left({n}_{rest}-1\right){ s}_{rest}^{2}+ \left({n}_{int}-1\right){ s}_{int}^{2}}{\left({n}_{rest}-1\right)+({n}_{int}-1)}}}$ for between-subject designs. To avoid overestimating the effect size, Hedges’ *g* was used (Cheung & Vijayakumar, 2016; Hedges, 1981). We used ${g}_{w}= d\times (1- \frac{3}{4\left({n}_{rest}-1\right)-1} )$ for within-subject designs and ${g}_{b}= d\times (1- \frac{3}{4\left({n}_{rest}+{n}_{int}-2\right)-1})$ for between-subject designs. Positive values indicated better memory in the rest condition relative to the interference condition and, conversely, negative values indicated worse memory in the rest condition. If data were reported in graphs in the original paper, but not in text or tables, data points were extracted with WebPlotDigitizer (Rohatgi, 2019).

Data were included from each study ranging from study characteristics, such as publication year and sample size, to experimental design, such as within versus between subjects. Descriptive statistics for included variables are shown in Tables 1 and 2. If a study included multiple delayed tests, only data from the first delayed test was used. If memory was measured in multiple ways for the same learning trial, data were included from the measures that assessed different aspects of memory. For example, if both recognition and free-recall tests were given, data from both tests were reported as separate effect sizes. However, when the measures reflected the same construct, one was selected (e.g., if both hits-false alarms and *d’* were reported, only *d’* was included). When studies included multiple conditions, effect sizes were calculated relative to the rest condition. For instance, if a rest period, an n-back task, and a spot-the-difference task were included, we reported two effect sizes, both relative to rest.

**Table 1:** Descriptive statistics for categorical variables

		Patients	Older adults	Adults
		*n*	*n*	*n*
**Design**^**1,2,3**^				
	within-	16	24	81
	between-		5	16
**Learning material**				
	word lists	7	14	44
	stories	7	9	12
	rating task^2^		1	17
	paired associates^1,2^	2	4	11
	routes^2^		1	8
	pictures^3^			1
	video^3^			4
**Learning modality**^**1,2**^				
	auditory	14	20	30
	visual verbal		5	40
	visual image		3	22
	visual auditory^2,3^	2	1	5
**Immediate test**^**1**^				
	yes	15	19	55
	no	1	10	42
**Interference task**				
	spot-the-difference	4	17	29
	working memory	7	8	54
	declarative memory^3^	5	4	7
	exercise^3^			1
	comprehension^3^			6
**Interference modality**^**1**^				
	auditory^2^	1	3	19
	visual auditory^3^			5
	visual image	4	17	46
	visual verbal	11	9	26
**Distractor**				
	yes	6	21	35
	no	10	8	62
**Testing method**^**1**^				
	joint	2	11	47
	separate	14	18	50
**Test type**^**1,2**^				
	free recall	16	24	53
	recognition		5	33
	cognitive map^3^			6
	cued recall^3^			5
**Test modality**^**1,2**^				
	verbal	16	24	39
	keyboard		4	33
	written		1	25
Due to insufficient effect sizes, certain predictors/levels were excluded from initial meta-regression analyses. These are denoted as:^1^ patients, ^2^ older adults, ^3^ adults

**Table 2:** Descriptive statistics for continuous variables

	Patients				Older adults				Adults			
	*M*	*SE*	Min	Max	*M*	*SE*	Min	Max	*M*	*SE*	Min	Max
**Publication year**	2012	1.5	2004	2021	2016	0.8	2005	2021	2017	0.5	2004	2024
**Rest interval (min)**	15.4	4.4	3	60	10.9	1.8	3	60	10.5	0.7	2	60
**Distractor interval (min)**	1.1	0.5	0	5	2.4	0.4	0	5	2.4	0.9	0	60
**Delay interval (min)**	17.6	4.3	9	60	362.3	347.1	9	10080	10239	1842.7	2	45284

Rest interval refers to the period immediately after learning and/or immediate testing, in which a participant either rests or is presented with an interference task. Distractor interval refers to any additional period after the rest period in which a distractor task is presented both to the rest and interference conditions. Finally, Delay interval refers to the total amount of time between learning and testing

In a within-subjects design, if the delayed memory tests for the two experimental conditions were given simultaneously, a rough estimate for delay interval (i.e., time-since-learning) score was calculated. For instance, for participants who were presented with the first set of learning material, asked to rest for 10 min, presented with the second set of learning material, asked to spot-the-difference for an additional 10 min, and then presented with memory tests for both sets of learning material, the delay interval was recorded as (10 min + 20 min)/2 = 15 min. Finally, we extracted the primary statistics provided by each study. For example, some studies used the memory performance change from immediate to delayed testing as their primary measure, while others used the delay scores. In each case, we followed what the authors considered their main finding.

### Analysis

#### Meta-analysis

A random-effects model was done to establish a more precise estimate of the wakeful rest effect. This model was selected because studies varied somewhat in their characteristics, and so a fair amount of between-study heterogeneity was expected. The model was estimated through the restricted maximum likelihood (REML) method (Viechtbauer, 2005) and the Knapp-Hartung adjustment was used to obtain the 95% confidence interval around the pooled effect (Knapp & Hartung, 2003).

Next, a moderator analysis was done to establish the influence of group differences (i.e., age and amnesia). Initially, there were five categories: patients, healthy older adults, adults, young adults, and children. However, because the younger and adult populations are so similar, the categories were combined. Only four datasets explored the effect on children and, thus, these were excluded from analyses.

An effect size was considered an outlier if its confidence interval did not overlap with the confidence interval of the pooled effect. Influential points were also examined using difference in fits (DFFITS), Cook’s distance (D), and hat values. An effect size was considered an influential point if one of three conditions was met: (1) $DFFIT{S}_{k}> \sqrt[3]{\frac{1}{k-1}}$, (2) ${D}_{k}$> 0.45, or (3) ${hat}_{k}>3\frac{1}{k}$.

#### Exploratory meta-regression

Given there are 14 potential predictors (Tables 1 and 2) and the absence of clear a priori hypotheses, the following exploratory approach was followed. First, each predictor was tested individually for its influence on the predicted effect size. For these initial models, REML and Knapp-Hartung adjustments were once again used. Predictors that demonstrated significant influence on the overall effect size were then included in a multiple meta-regression. For these models, ML was used. A step-wise procedure was used, in which predictors were removed based on the amount of heterogeneity explained in individual models. This process was carried out separately for each group (patients, older adults, and adults) because there was large heterogeneity between groups. All continuous variables were mean-centered.

## Results

### Meta-analysis

Based on the random-effects model, the overall pooled effect size was *g* = 0.34 (95% CI: 0.26–0.43; *p* <.0001). The estimated between-study heterogeneity was *τ*^*2*^ = 0.13 (95% CI: 0.12–0.28), with *I*^*2*^ = 63.0% (95% CI: 55.7–69.1), indicating moderate to large heterogeneity. The prediction interval of the effect size ranged from *g* = −0.37 to 1.06, indicating that negative intervention effects cannot be ruled out for future studies. The test for group differences (i.e., age and amnesia) was significant (*p* <.0001). Results for each group are described below.

### Patients

**Fig. 3:**
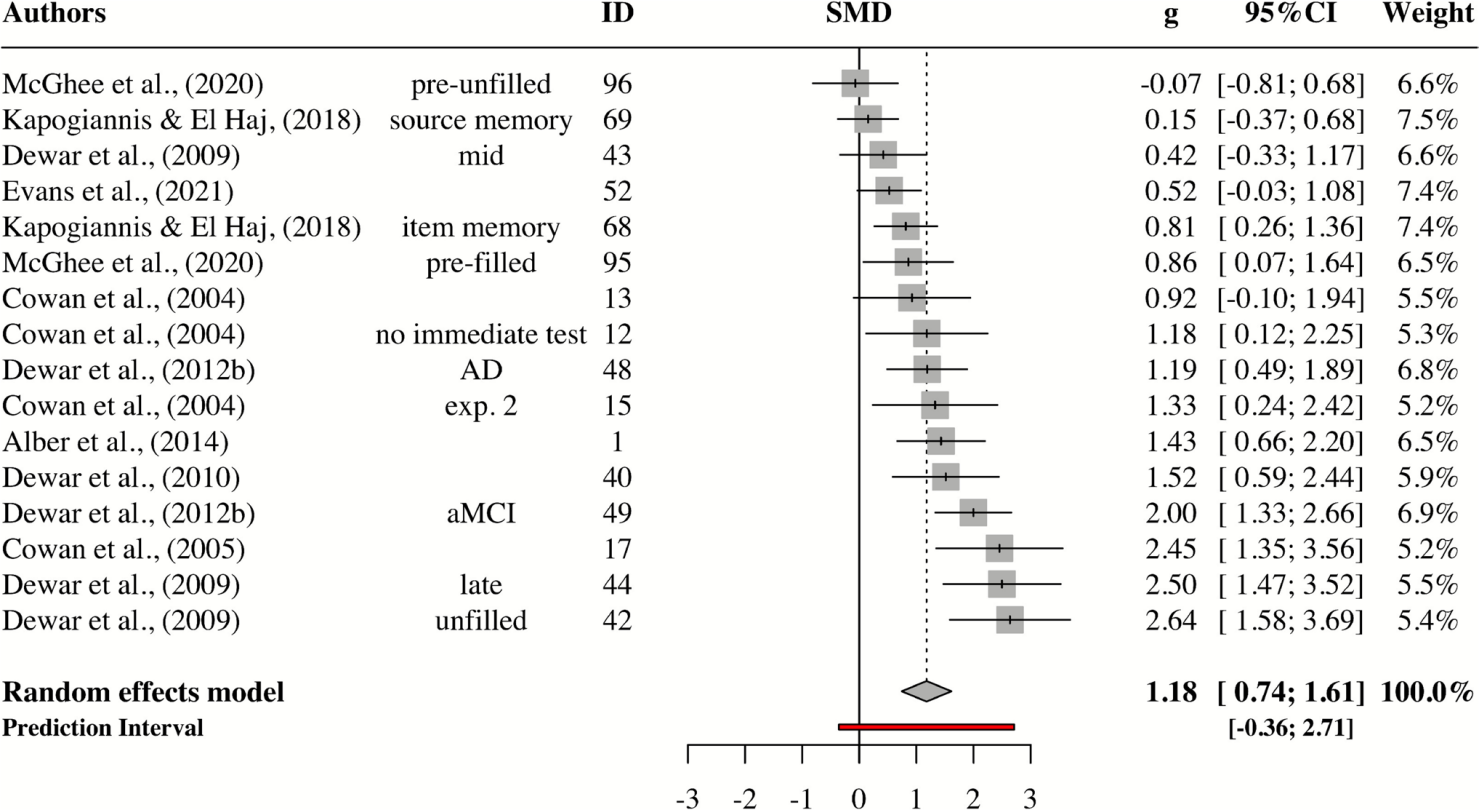
Forest plot for patients with anterograde amnesia

For patients, the pooled effect size was very large, *g* = 1.18; 95% CI: 0.74–1.61; p <.0001 (Fig. 3). The estimated between-study heterogeneity was *τ*^*2*^ = 0.48 (95% CI: 0.18–1.43), with *I*^*2*^ = 74.7% (95% CI: 58.8–84.5). Thus, we can assume moderate to large heterogeneity. The prediction interval ranged from *g* = −0.36 to 2.71, indicating that negative intervention effects cannot be ruled out for future studies.

**Fig. 4:**
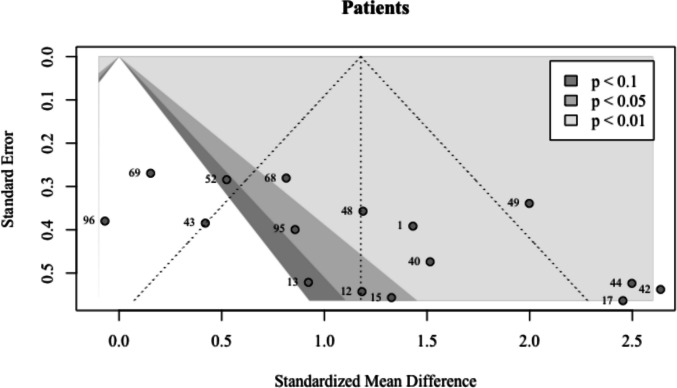
Funnel plot for patients. The points represent 16 patient effect sizes. Those that lie within the two lightest gray areas are statistically significant (*p* < 0.05 or *p* < 0.01). Egger’s regression test indicates asymmetry in the plot

Two outliers were found (Kapogiannis & El Haj, 2018 #69 and McGhee et al., 2020 #96), and removing them had almost no impact on the pooled effect (*g* = 1.35; 95% CI: 0.93–1.76; *p* <.0001). The between-study heterogeneity was reduced to *τ*^*2*^ = 0.32 (95% CI: 0.08–1.16), along with a reduced* I*^*2*^ = 65.1% (95% CI: 38.4–80.2). In addition, the prediction interval now only displayed positive values, indicating a more robust pooled effect (*PI*: 0.06–2.63). No effect sizes met any of the three criteria for influential points. Egger’s regression test (Egger et al., 1997) was significant for asymmetry (${\widehat{\beta }}_{0}$= 4.69; *t* = 2.87; *p* =.01), suggesting some evidence for publication bias (Fig. 4).

### Older adults

**Fig. 5:**
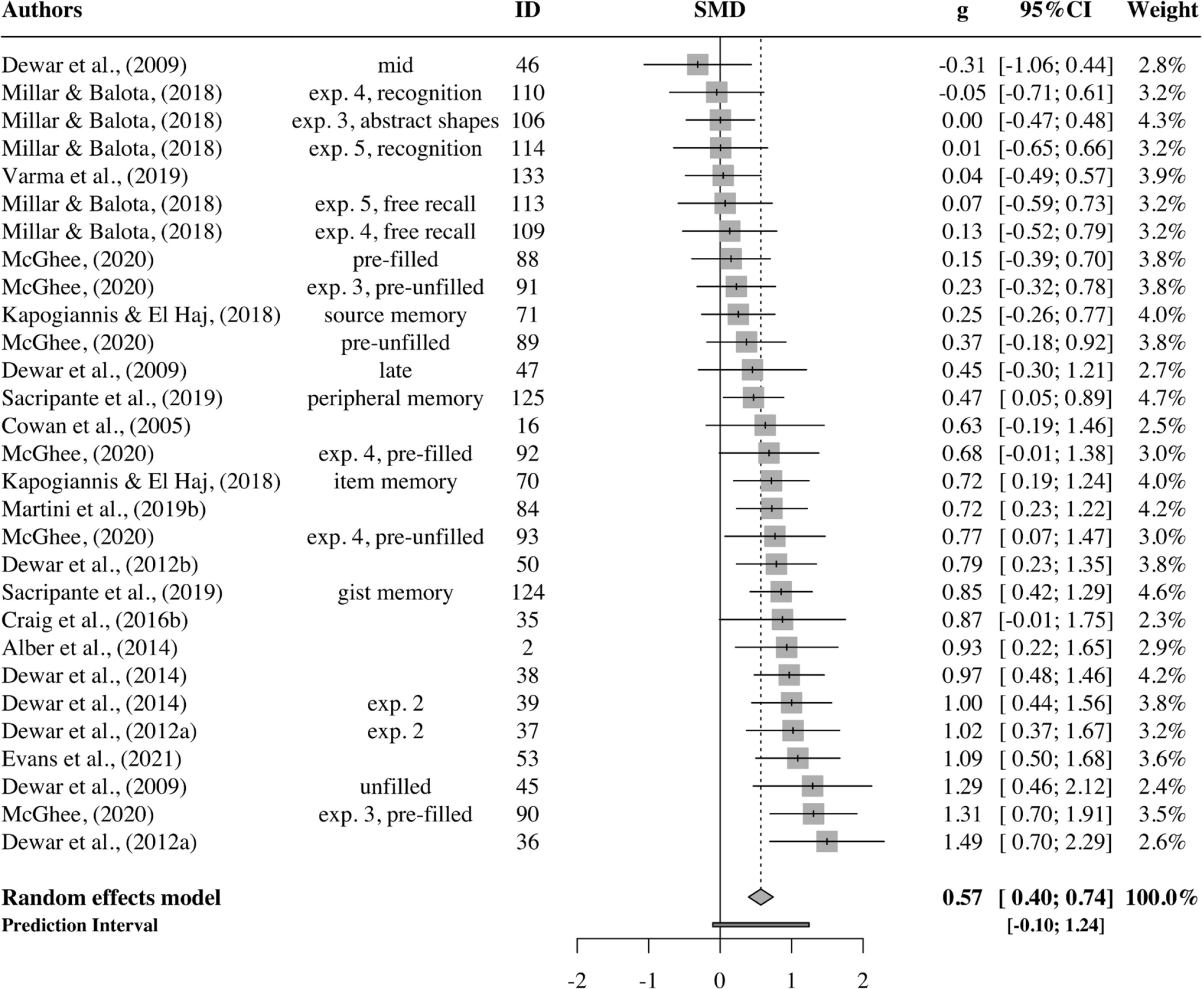
Forest plot for healthy older adults

For older adults, the pooled effect size was smaller than that for patients, *g* = 0.57; 95% CI: 0.40–0.74; *p* <.0001 (Fig. 5). The estimated between-study heterogeneity was *τ*^*2*^ = 0.10 (95% CI: 0.03–0.29), with *I*^*2*^ = 52.4% (95% CI: 27.2–68.8). Thus, we can assume moderate heterogeneity. The prediction interval ranged from *g* = −0.10 to 1.24, indicating that negative intervention effects cannot be ruled out for future studies.

**Fig. 6:**
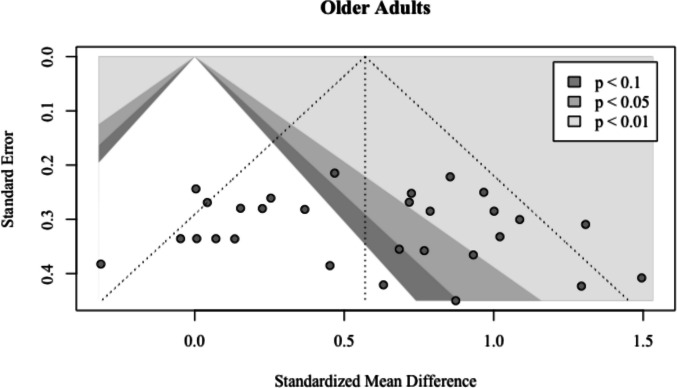
Funnel plot for older adults. The points represent 29 older adult effect sizes. Those that lie within the two lightest gray areas are statistically significant (*p* < 0.05 or *p* < 0.01)

No outliers or influential points were found and Egger’s regression test was not significant for asymmetry (${\widehat{\beta }}_{0}$ = 0.89; *t* = 0.62; *p* =.54), suggesting no evidence for publication bias (Fig. 6).

### Adults

**Fig. 7:**
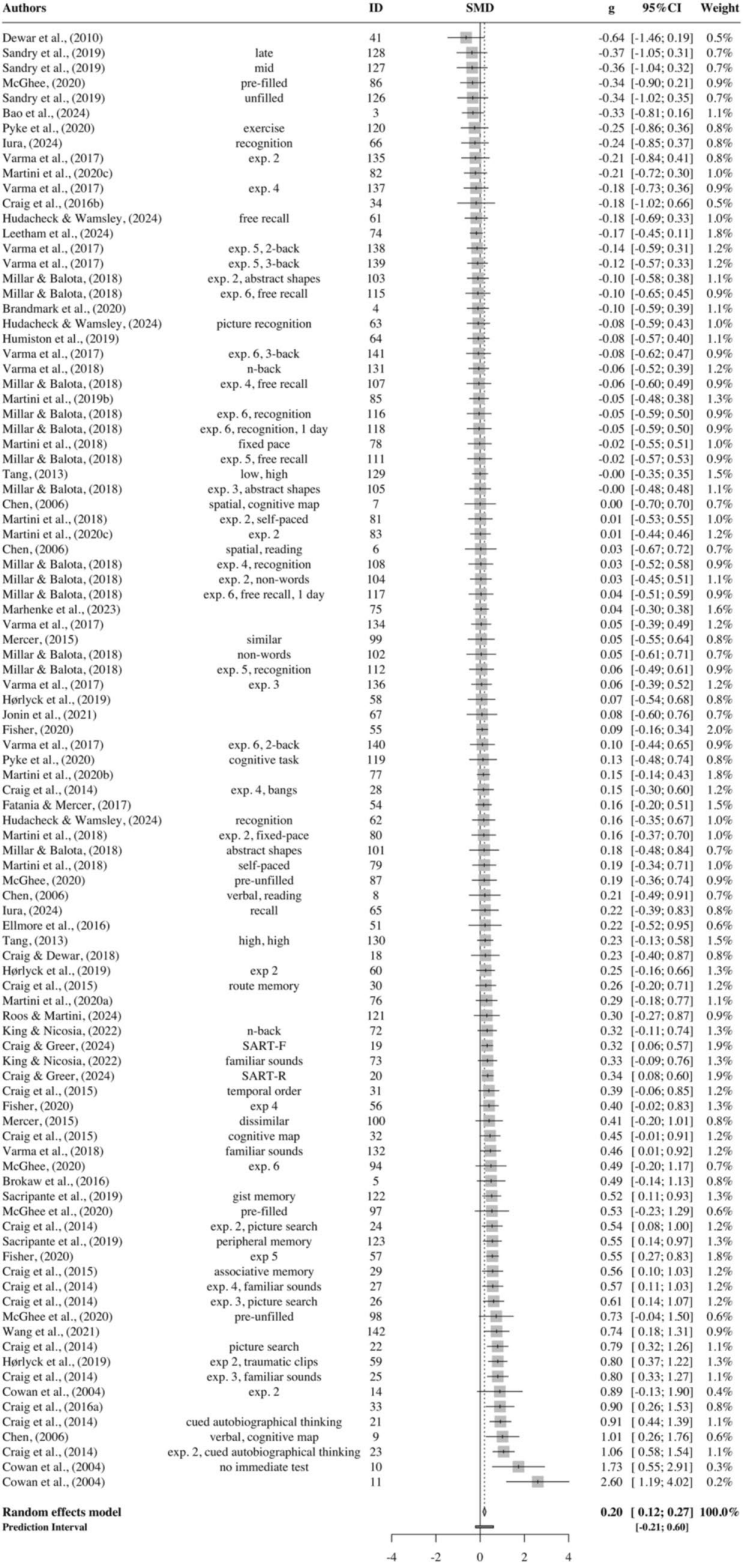
Forest plot for healthy young and middle-aged adults

As expected, the pooled effect size was the smallest for adults, *g* = 0.20; 95% CI: 0.12–0.27; *p* <.0001 (Fig. 7). The estimated between-study heterogeneity was *τ*^*2*^ = 0.04 (95% CI: 0.03–0.11), with *I*^*2*^ = 44.0% (95% CI: 28.7–56.1). Thus, we can assume moderate heterogeneity. The prediction interval ranged from *g* = −0.21 to 0.60, indicating that negative intervention effects cannot be ruled out for future studies.

**Fig. 8:**
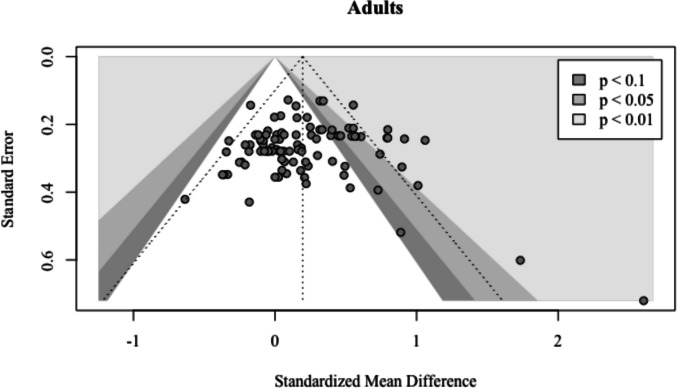
Funnel plot for adults. The points represent 97 adult effect sizes. Those that lie within the two lightest gray areas are statistically significant (*p* < 0.05 or *p* < 0.01)

Nine outliers were found (Cowan et al., 2004 #10; Cowan et al., 2004 #11; Craig et al., 2014 #21; Craig et al., 2014 #22; Craig et al., 2014 #23; Craig et al., 2014 #25; Fisher, 2020 #57; Hørlyck et al., 2019 #59; Leetham et al., 2024 #74). Removing them resulted in a smaller, but still significant and positive, pooled effect (*g* = 0.15; 95% CI: 0.09–0.21; *p* <.0001). The between-study heterogeneity was substantially reduced to *τ*^*2*^ = 0.00 (95% CI: 0.00–0.04), along with a reduced *I*^*2*^ = 10.5% (95% CI: 0.0–32.0). The prediction interval narrowed and no longer predicted negative intervention effects (*PI*: 0.01–0.29). Once again, no influential points were found and Egger’s regression test was not significant for asymmetry (${\widehat{\beta }}_{0}$= −0.35; *t* = 0.56; *p* =.58), suggesting no evidence for publication bias (Fig. 8).

### Exploratory meta-regression

#### Patients

For patients, only publication year was significant based on the analysis with just one moderator. The predicted effect for a study published in 2012, the average publication date, was *g* = 1.22 (95% CI: 0.85–1.59; *p* <.0001). For every additional year, the effect size was expected to decrease by 0.08 (95% CI: −0.14–0.01; *p* =.02). Publication year explained ${R}_{*}^{2}=$ 42.6% of heterogeneity for studies including patients. None of the other predictors significantly influenced effect size differences for patients.

Thus, the final model only included publication year. This model fit the data significantly better than a random-effects model with no predictor, ${\chi }_{1}^{2}$ = 6.46, *p* = 0.01. With the inclusion of publication year, unexplained between-study heterogeneity was reduced from *I*^*2*^ = 74.0% to 58.1%. The amount of heterogeneity accounted for was ${R}_{*}^{2}$ = 51.2%.

#### Older adults

For older adults, immediate test explained a significant amount of heterogeneity (${R}_{*}^{2}$ = 19.7%). Including a memory test directly after learning resulted in a larger predicted effect size (*g* = 0.70; 95% CI: 0.49–0.90; *p* <.0001) than not including one (*g* = 0.35; 95% CI: 0.08–0.62; *p* =.01), *F*(1,27) = 4.53, *p* =.04. Interference task also explained a large portion of heterogeneity (${R}_{*}^{2}$= 30.9%). The predicted effect size for a study using spot-the-difference tasks (*g* = 0.70; 95% CI: 0.50–0.90; *p* <.001) was significantly larger than for those using working memory tasks (*g* = 0.30; 95% CI: 0.02–0.59; *p* =.04), *F*(1, 23) = 5.52, *p* =.03. None of the other predictors influenced effect size differences for older adults.

Immediate test and interference task were then included in a multiple meta-regression. The full model fit the data significantly better than a reduced model without immediate test, ${\chi }_{1}^{2}$ = 4.67, *p* = 0.03. With the inclusion of immediate test and interference task, unexplained between-study heterogeneity was reduced from *I*^*2*^ = 50.8% to 30.2%. The amount of heterogeneity accounted for was ${R}_{*}^{2}$ = 57.5%.

#### Adults

Learning modality explained a significant amount of heterogeneity (${R}_{*}^{2}$ = 27.2%, *F*(1,89) = 5.95, *p* =.004). The predicted effect size for studies presenting auditory learning materials, in which people listened to the material to-be-remembered (*g* = 0.38; 95% CI: 0.26–0.51; *p* <.0001) was larger than for studies that had people read the learning material (*g* = 0.13; 95% CI: 0.03–0.22; *p* = 0.009), *t*(89) = 3.21, *p* =.002, and for studies in which people were presented with visual images (*g* = 0.12; 95% CI: −0.03–0.26; *p* =.11), *t*(89) = 2.77, *p* =.007. There was no difference between the latter two, *t*(89) = 0.02, *p* =.09.

The presence or absence of a distractor task after the rest or interference interval also explained a large amount of heterogeneity (${R}_{*}^{2}$= 11.5%). The predicted effect size for studies with a distractor task (*g* = 0.07*;* 95% CI: −0.05–0.19; *p* =.22) was smaller than for those without (*g* = 0.26; 95% CI: 0.17–0.34; *p* <.0001), *F*(1, 95) = 6.28, *p* =.01.

Memory test type explained ${R}_{*}^{2}$ = 7.6% of heterogeneity. The predicted effect size for studies using free recall (*g* = 0.24; 95% CI: 0.14–0.33; *p* <.0001) was larger than for studies using recognition (*g* = 0.08; 95% CI: −0.04–0.20; *p* =.17), *F*(1, 84) = 3.93, *p* =.05. Testing modality explained ${R}_{*}^{2}$ = 19.2% of heterogeneity (*F*(1, 94) = 4.05, *p* =.02). The predicted effect for a study in which participants responded verbally during the memory test (*g* = 0.32; 95% CI: 0.21–0.44; *p* <.0001) was larger than keyboard/mouse responses (*g* = 0.09; 95% CI: −0.02–0.21; *p* =.11), *t*(94) = 2.82, *p* =.006, but there was no difference with written tests (*g* = 0.18; 95% CI: 0.05–0.30; *p* =.005), *t*(94) = 1.73, *p* =.09. There was also no difference between keyboard/mouse responses and written tests, *t*(94) = 0.99, *p* =.32.

While delay interval initially explained a significant amount of variance (${R}_{*}^{2}$= 30.6, *F*(1, 95) = 11.52, *p* =.001), this disappeared when studies with delays of 1 day or longer were removed (${R}_{*}^{2}$= 16.0%, *F*(1, 65) = 1.86, *p* =.18). Finally, publication year was a significant predictor. The predicted effect for a study published in 2017, the average publication date, was *g* = 0.22 (95% CI: 0.12–0.25; *p* <.0001). For every additional year, the effect size was expected to decrease by 0.03 (95% CI: ˗0.05 – ˗0.01; *p* =.001). Publication year explained ${R}_{*}^{2}=$ 12.9% of heterogeneity for studies including healthy adults. None of the other predictors significantly influenced effect size differences for adults.

Learning modality, distractor, test type, test modality, and publication year were then included in a multiple meta-regression. Thirty-three of the 39 verbal tests were free recall, 31 of the 33 keyboard tests were recognition, and 20 of the 25 written tests were free recall. Due to the lack of variability and because test type explained less heterogeneity, test type was not included. The remaining model fit the data significantly better than a reduced model without distractor task, ${\chi }_{1}^{2}$ = 4.74, *p* = 0.03. With this final model, unexplained between-study heterogeneity was reduced from *I*^*2*^ = 39.8% to 25.2%.

## General discussion

The aim of these analyses was to pool data from numerous studies and evaluate the evidence for the wakeful rest effect with a particular focus on identifying the influence of age and anterograde amnesia on the post-learning wakeful rest benefit. This was done because the literature has inconsistent results for young to middle-aged healthy adults. Meta-regression analyses were also done, with the aim of identifying the influence of a variety of study characteristics and accounting for unexplained between-study heterogeneity.

A total of 142 effect sizes from 51 different experimental studies were identified. Results of the meta-analysis suggest that a short period of wakeful rest after learning is beneficial for all groups; patients with anterograde amnesia, healthy older adults, and healthy young to middle-aged adults. Moderator analyses subsequently showed a significant effect of age and amnesia, indicating differences in effect size across groups. The between-study heterogeneity was quite large, suggesting that the groups should be analyzed separately. Thus, studies were further divided into age and amnesia groups; namely, 16 effect sizes representing patient populations, 29 for healthy older adults, and 97 for healthy young to middle-aged healthy adults.

### Meta-analytic summary

Unsurprisingly, patient studies produced the largest combined wakeful rest effect. Studies with healthy older adults also produced a large pooled effect, although it was notably smaller. Finally, and as expected, studies involving young and middle-aged adults produced the smallest, but still significant, pooled effect.

These findings are in line with the hypothesis that patients with anterograde amnesia benefit the most from reduced interference after learning. It follows that older adults, who have stronger memories than patients with amnesia, do not benefit to the same extent. However, because they often demonstrate weaker memory performance than younger and middle-aged adults, it was reasonable to expect that they would benefit more from resting after learning. Finally, despite having a small pooled effect, young and middle-aged adults still showed some benefit from resting after learning. Thus, the current results support the idea that memory efficiency modulates the effectiveness of a short period of rest on improving memory.

As evidenced by the meta-regression, a variety of study characteristics also seem to impact this benefit. For studies with patients and younger adults, publication year was a significant moderator of the wakeful rest effect. Earlier studies across both groups produced a larger effect, with a decreasing effect size over time. For patients, this is in line with the fact that Egger’s regression test suggested the presence of publication bias. Below, we discuss two possible explanations for this finding.

First, patient studies are notoriously under-powered, and these small samples can affect the variable results found overtime. Of the studies included here, patient studies had the smallest sample sizes (*M* = 14, *SE* = 1.78) compared to healthy older adult studies (*M* = 22, *SE* = 1.69) and younger to middle-aged adults (*M* = 33, *SE* = 2.32). Moreover, other study characteristics, such as interference task, appear to be strongly associated with year of publication, reflecting a shift in materials used by researchers. For instance, earlier studies used working memory tasks in a visual verbal task modality and did not include a distractor task before testing, while later studies used spot-the-difference tasks in visual image modalities and did include a pre-testing distractor task. As such, it is unclear which of these methodological aspects is driving the effect.

Interestingly for younger adult studies, publication year does not appear to be clearly related to other study characteristics. This suggests that publication year may serve as a proxy for a study characteristic that was not accounted for in the current analysis. In any case, Egger’s regression test found no evidence for asymmetry in funnel plots for younger adult studies, indicating that publication bias is likely not the issue. For both groups, the model with the best fit included publication year as a predictor.

Immediate test and interference task explained significant portions of variability for healthy older adults. Including a memory test directly after learning and before the rest or interference interval resulted in a larger predicted effect size than not including one. However, the estimated effect was significant regardless of whether an immediate test was included. These results indicate that testing effects are present and suggest that the memory benefits of rest may be enhanced if paired with an immediate test.

For interference tasks, healthy older adult studies with spot-the-difference tasks generated significantly larger estimated effects than working memory tasks. These findings are inconsistent with the consolidation theory of wakeful rest, which posits that observed memory benefits are driven by active neural processes occurring during wakeful rest, not by disruptive processes occurring during the interference period. Theoretically, no differences arising from interference tasks are anticipated. However, it would be overly simplistic to assume that the interference interval does not contribute to memory disruption to some degree. In this regard, it follows that people with weaker memory abilities, such as older adults, may be more susceptible to post-learning interference, thereby contributing to the variability in the effectiveness of rest. However, it is unclear why interference task did not seem to be a significant influence of the wakeful rest effect for patients. It could be that any kind of interference contributes to disruption of consolidation processes.

Variation in effect sizes is not explained by interference tasks for younger adults, in line with consolidation theory. This provides some support that neural processes are actively encouraging memory consolidation during rest.

However, learning modality did significantly influence the effectiveness of rest for younger adults. Studies that presented the learning material auditorily showed the largest pooled effect, followed by visual verbal (i.e., reading), and then visual image modalities. These correspond to the types of learning materials used; many word lists and stories were presented auditorily or read by the participants, whereas rating tasks were primarily visual images. Distractor task was also significant for younger adults. The predicted wakeful rest effect is not significant when a distractor task is included, but it is when it is not. Although previous work has suggested that a period of post-encoding rest is beneficial regardless of eventual interference (Dewar et al., 2009), this result suggests that a distractor task leads to some disruption of any rest benefit. That said, it is unclear why distractor task did not seem to influence patient and older adult outcomes.

Test type and test modality were not analyzed for patients and older adult studies because most of these studies used verbal free recall. For adult studies, pooled effects were large when participants responded verbally, but small and non-significant when making mouse clicks or key presses. Written tests also resulted in a significant prediction. Verbal memory tests consisted predominantly of free recall (with the exception of six datasets). Similarly, 20 of the 25 written tests were free recall while all but two keyboard tests were recognition. In line with this finding, free-recall tests resulted in a significant expected effect, while recognition tests did not. There is an extensive literature suggesting that different brain regions contribute to free recall and recognition (Yonelinas, 2002), which may influence these disparities.

## Limitations

While these findings provide important insights into the effectiveness of wakeful rest in improving memory, there are a number of limitations. First, there was moderate to large between-study heterogeneity among studies of all groups. This is not surprising, given that studies differed in a variety of study characteristics, including learning materials, interference tasks, and memory tests. However, heterogeneity can be due to a number of reasons. For instance, patient studies produced the largest between-study heterogeneity. This could indicate that a substantial proportion of the variation in the data stems from true effect size differences. In the context of patient studies, a variety of etiologies for amnesia were represented in the dataset, including, but not limited to, MCI, Alzheimer’s disease, and embolic strokes (Cowan et al., 2004; Dewar et al., 2012b; Evans et al., 2021). Another explanation is small sample sizes. Patient studies had as few as six participants per experiment. This also explains why standard errors around effect sizes were large (Fig. 4).

However, the results after the removal of two outliers suggested that patient studies are unlikely to produce nonexistent or detrimental memory outcomes of post-learning rest. The overall effect increased, providing more confidence in its robustness. Moreover, between-study heterogeneity decreased, albeit slightly. It was further reduced with the inclusion of publication year in the model.

Older adult studies showed less heterogeneity across studies. The inclusion of two predictors, immediate test and interference task, reduced the between-study heterogeneity to low levels. Adult studies showed less heterogeneity than both patient and older adult studies. Heterogeneity decreased considerably to a small percentage of between-study variation with the removal of nine outliers.

Another limitation is that a step-wise approach was used for the meta-regression. Although this approach is frequently used, it has important disadvantages. Namely, it may produce biased estimates and there are multiple hypothesis testing issues (Whittingham et al., 2006). Therefore, the current findings should be viewed as preliminary and interpreted carefully. The aim of this meta-regression was to provide a guide for future research, with the results serving as a foundation for empirical testing.

## Future directions

Despite these limitations, this study has notable strengths. Although it is not the first meta-analysis of wakeful rest, it is the first to explore the influence of age and amnesia, as well as study characteristics, such as learning material, interference task, and memory test. The current study provides comprehensive evidence for a more robust understanding of the effectiveness of short periods of rest in boosting memory. Moreover, many studies were included, leading to increased statistical power and the identification of a more precise estimate of the wakeful rest effect. This was particularly important given that the current literature is inconsistent.

Outstanding questions remain, and these findings can be used to plan future research. For example, most studies focused on short delays that ranged from 9 min to 8 days. Previous research shows that after about a week, a shift exists in memory, and the pattern of forgetting changes (Fisher & Radvansky, 2018). Thus, to take full advantage of rest-related memory improvements, future work should explore how rest-related memory changes over time, beyond a 1-week delay.

In another set of studies, the impact of study characteristics should be explored. Here, quite a few study characteristics explained effect size variation. These should be tested empirically. There could be differences in how people across age groups encode simple versus complex material. Moreover, research should be implemented investigating how different levels of memory representation are affected by a short period of rest; and whether rest supports consolidation for surface-level information and how that differs from more complex information, such as event models.

Future research informed by these findings could also investigate which kinds of tasks interfere with rest-related consolidation and which are equivalent to wakeful rest, potentially ones that are more likely to engage the default mode network, which is a collection of brain regions that is active when people are at rest.

On a related note, future research should explore whether stress and anxiety modulate the quality of rest after learning. The current findings suggest that wakeful rest is not as effective for improving memory for younger adults as it is for older adults. In preliminary experiments that we have carried out exploring the effect in younger adults, it became clear that many participants were not resting during the rest periods. Instead, the students asked if they were allowed to do homework or use their phones. In addition, many were visibly annoyed or impatient at the instruction to relax and sit quietly for 10 min. Younger adults, often college students, typically find themselves burdened by schoolwork and other extracurriculars, which can make it difficult to relax. A potential explanation for the smaller effects observed here is that younger adults are not engaging the default mode network. That said, Hudachek and Wamsley (2024) found that in young adults, higher anxiety scores were associated with a greater wakeful rest effect for emotional stimuli. While heart rate was recorded, this (or any other physiological measure of stress/anxiety) was not reported during the rest or task period. To assess the true magnitude of the wakeful rest effect, it is crucial to evaluate the rest quality younger adults experience. Thus, it is important to understand the role stress and anxiety play in the ability to rest.

Moreover, in daily life, consolidation processes occur even throughout the busiest, interference-filled days; otherwise, we would not be able to remember what we had for breakfast by the end of the day. It is unknown if and how interpolated bursts of rest during learning allow that to occur.

Finally, the current results suggest that wakeful rest improves memory across age and amnesia groups, but does it also improve other cognitive abilities? Sleep research has found a relationship between consolidation and creativity (Lewis et al., 2018), which should be explored using wakeful rest.

## Conclusion

This line of research on wakeful rest tells us when rest is beneficial and why. Promising results in amnesic patient populations have already been shown, in that even they benefit from short periods of rest. A deeper understanding of this phenomenon holds the potential for clinical interventions for people with different kinds of memory-related disorders. Moreover, rest-related improvements in memory have implications to aid people in their day-to-day life, in school, and in work, and can even inform interventions to protect against age-related memory decline. The current study provides a broad assessment of the studies that have been done to date and assesses the circumstances that magnify or diminish this effect. This meta-analysis provides the basis for designing more effective and targeted studies of wakeful rest. In sum, maybe we *should* all just take 10.

## Data Availability

All data and relevant code are available at https://osf.io/jmvgf/overview.

## Footnotes

1 Although some of the participants were older adults, the majority were not. As such, we decided to report this finding in this section.

2 Studies were screened by the first and last authors. Classifications and coding decisions were made by all three of the authors working in collaboration.

## Code availability

All data and relevant code are available at https://osf.io/jmvgf/overview.

## Declarations

### Open practices statement

An analysis plan was preregistered and, along with any deviations, is available at https://osf.io/jmvgf/overview. All data and relevant code have also been made publicly available there.
